# Dataset of allelopathic effects of *Casuarina equisetifolia*-L leaf aquatic extract on seed germination and growth of selected plant crops

**DOI:** 10.1016/j.dib.2019.104770

**Published:** 2019-11-07

**Authors:** Talaat A. Ahmed, Ahmed Abou Elezz, Noura H. Al-Sayed

**Affiliations:** a Environmental Science Center (ESC), Qatar University, P.O. Box 2713, Doha, Qatar; b Department of Biological and Environmental Sciences, College of Arts and Sciences, Qatar University, Doha, 2713, Qatar

**Keywords:** *Casuarina equisetifolia*-L, Plant crops, Seed germination, Seedling growth, Greenhouse, Inhibition

## Abstract

In agricultural fields that are surrounded by dunes, the *Casuarina* tree is usually used as windbreaks. In addition, it plays a significant role in inhibiting nearby vegetation growth. In this dataset, the effect of *Casuarina equisetifolia*-L leaf aquatic extract on seed germination and growth of four selected plant crops (Maize, Lentil, Mustard, and Wheat) was tested. Seed germination test and a greenhouse experiment were conducted to study the effects of *Casuarina equisetifolia* aquatic leaf extract on seed germination and seedling growth. Various concentrations of leaf extract were used (0, 1.25%, 2.5%, and 5%). Results revealed that aqueous leaf extract of *Casuarina equisetifolia*-L affected significantly seed germination of all selected plant crops under laboratory conditions.

Both wheat and maize have a comparable reduction in root length at 2.47 cm and 2.45 cm, respectively, but show a remarkable difference in percentage at 92% and 54% respectively under the effect of 5% aqueous solution. Similarly, lentil shows the highest drop in shoot length (SL) of 69% while no significant reduction observed in Mustard (SL) at the same aqueous solution concentration (5%).

In the greenhouse experiment, dried leaf powder was mixed with soil in different concentrations (0, 15, 30, and 45 g kg^−1^ soil). Greenhouse experiments demonstrated that *C. equisetifolia* exhibited significant inhibition on wheat germination and seedling characteristics under all treatments. However, soil pH and electrical conductivity (EC) were examined and showed a relative reduction in pH and marked an increase in (EC).

**Table:** Specifications Table

Subject	Agricultural, chemical science
Specific subject area	Allelopathic effect of *Casuarina equisetifolia* leaf on seed germination
Type of data	Table, figure, chart
How data were acquired	Different leaf aquatic extract concentrations and leaf powder were used to study the effect of *Casuarina equisetifolia* leaf on seed germination, and data were analyzed using SPSS Package 25 and MS Excel 2016.
Data format	Raw, analyzed
Parameters for data collection	Seed germination percentage (GP), root and shoot length of selected plant crops were investigated in the lab greenhouse under different concentrations of *Casuarina* dried leaf. The pH and Electrical Conductivity (EC) of soil were tested using jenway pH and conductivity meter.
Description of data collection	A four plant crops (Maize, lentil, Mustard, and Wheat) were tested for germination with different concentrations (0, 1.25%, 2.5%, and 5%) of leaf extract and (0, 15, 30 and 45 g kg^−1^) of well-dried leaf powder in the greenhouse were mixed with soil to study the seedlings inhibition effect during the study period.
Data source location	Institution: Environmental Science Center (ESC) and Department of Biological and Environmental Sciences (DBES), Qatar University City: Doha Country: Qatar Latitude and longitude: (25°48′27.70″N, 51°20′47.10″E)
Data accessibility	The raw data was archived in Mendeley Data https://data.mendeley.com/datasets Reserved https://doi.org/10.17632/g8jvhmxy9h.4 Link to the preview https://data.mendeley.com/datasets/g8jvhmxy9h/4

**Table:** 

**Value of the Data** • Understanding the allelopathic effects of *Casuarina equisetifolia*-L on some plant crops (Maize, lentil, Mustard, and Wheat) germination percentage and seedling inhibition, root and shoot length. • The present dataset could be useful for concerned researchers and those who interested in cultivation, from the potential effects of crops surrounded by *Casuarina* trees in order to select the candidate most suitable plant. • Furthermore, the dataset introduced in this article can be used to investigate the possible effect of the chemicals released from *Casuarina equisetifolia* leaves on the potential of hydrogen (pH) and Electrical Conductivity (EC) of the soil.

## 1 Data

Allelopathy refers to the valuable or damaging effects of a specific plant on another, both crop and weed species, by the release of chemicals from plant parts [1]. Phenolics, terpenoids, and organic cyanides were reported as active allelochemicals can be leaching from leaves and stem of *Casuarina* trees and many plants to the environment [[2], [3], [4]]. The current data focused on the potential effects of *Casuarina equisetifolia*-L on Maize, lentil, Mustard, and Wheat crops during the study.

An experiment was conducted to measure the germination percentage, seedling inhibition, root and shoot length under the effect of the allelochemical compounds released from *Casuarina equisetifolia* leaves on Maize, Lentil, Mustard, and Wheat crops using Petri dishes under controlled lab conditions.

The second experiment was performed using pots in the greenhouse, to study the actual effect of the fallen *Casuarina equisetifolia* leaves on the wheat crop germination and seedling in the presence of Mustard as a weed. Furthermore, the effect of soil mixed with *Casuarina equisetifolia-L* powder on (pH), and Electrical Conductivity (EC) were studied.

### 1.1 Petri-dish experiment

An aqueous leaf extract solution of *Casuarina equisetifolia*-L was added to each Petri dish to study the potential inhibition effects of germination in all crops selected. Significant effects on seeds germination were observed in the laboratory. Seedling growth results from the current study showed that the germination percentage, shoot, and root length of lentil, wheat, mustard, and maize were markedly suppressed by increasing the leaf aqueous extract concentration. (Table 1, Table 2).

**Table 1:** Means of seed germination percentage, shoot length, root length (cm), and standard deviation as influenced by different concentrations of *Casuarina* leaf extracts of Mustard and Maize.

Treatment	Mustard						Maize					
	GP	±SD	RL	±SD	SL	±SD	GP	±SD	RL	±SD	SL	±SD
Control	95	14.8	3.51	1.11	2.03	0.34	72.5	5	4.51	1.84	1.65	0.46
1.25%	85	14.8	3.21	0.57	2.32	0.8	52.5	12.5	5.03	0.94	2.26	0.32
2.50%	90	8.6	2.66	0.82	2.54	0.47	60	8.16	3.52	0.5	1.82	0.36
5%	66.67	12.2	1.56	0.71	1.97	0.77	37	5	2.06	0.25	1.43	0.13

Germination percentage (GP), Standard Deviation (SD), root length (RL), shoot length (SL).

**Table 2:** Means of seed germination percentage, shoot/root length (cm), and standard deviation as influenced by different concentrations of *Casuarina* leaf extracts of lentil and Wheat.

Treatment	lentil						Wheat					
	GP	±SD	RL	±SD	SL	±SD	GP	±SD	RL	±SD	SL	±SD
Control	100	0	2.21	0.52	1.91	0.15	95.00	4.08	2.68	0.8	2.29	0.13
1.25%	92.5	9.57	1.57	0.37	1.05	0.24	87.50	6.45	2.11	0.57	2.15	0.24
2.50%	95	5.77	1.15	0.22	0.76	0.14	78.75	2.50	0.25	0.16	1.62	0.12
5%	80	8.16	0.66	0.03	0.59	0.13	67.50	2.89	0.21	0.13	1.26	0.33

Germination percentage (GP), Standard Deviation (SD), root length (RL), shoot length (SL).

#### 1.1.1 Descriptive data

One-way ANOVA test was done to check the growth variation between four different treatments (0, 1.25, 2.5, and 5%) in the laboratory. A significant variation in (GP) was observed between the treatments in Lentil, Mustard, and Maize while in Wheat no significant difference found (Table 3).

**Table 3:** Analysis of variance of seed germination percentage as influenced by different concentrations of *Casuarina* leaf extracts of Mustard, Maize, Lentil, and Wheat.

Source of Variation	DF	MS											
		Mustard			Maize			Lentil			Wheat		
		GP	SL	RL	GP	SL	RL	GP	SL	RL	GP	SL	RL
Between	3	611.2^a^	0.28	2.95^a^	856.25^a^	0.49^a^	6.84^a^	289.58^a^	1.36^a^	1.72^a^	559.90^a^	0.90^a^	0.35^a^
Within	12	164.8	0.34	0.57	68.75	0.11	1.14	47.92	0.02	0.11	18.23	0.04	0.24
Total	15												

a Indicates significant differences among means at 0.05. MS; Means of squares. Germination percentage % (GP%), shoot length (SL), root length (RL).

Shoot and root length variations were tested under the same treatments and conditions.

Significant variation was recorded between treatments in all seeds except Mustard shoot length (*p* > *0.05*) at a confidence interval of 95% (Table 3).

**Fig. 1:**
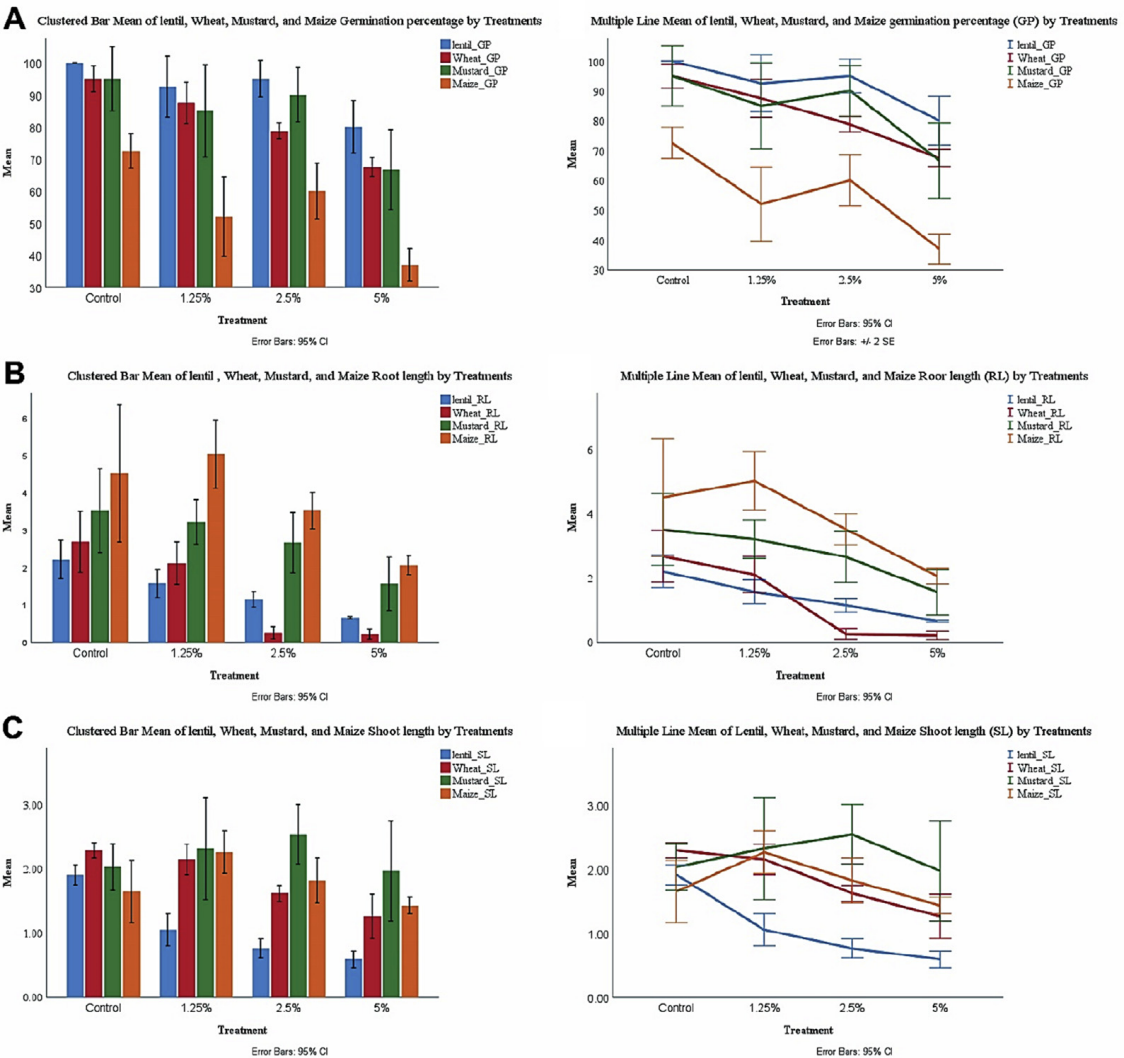
The mean measurement data of germination percentage (GP), Root, and Shoot lengths (cm) for four crops under controlled lab conditions. (A)Mean germination %, (B)Mean root length, (C)Mean shoot length.

The mean values of germination% (GP), shoot length (SL), and root length (RL) (with a standard deviation) for the studied crops were illustrated in Fig. 1

### 1.2 Greenhouse experiment

Greenhouse experiments were conducted to investigate the potential effect of *Casuarina equisetifolia* leaves on seedling growth of Wheat as a crop and Mustard as a weed with four treatments (control, 15, 30, 45 g/kg soil).

**Fig. 2:**
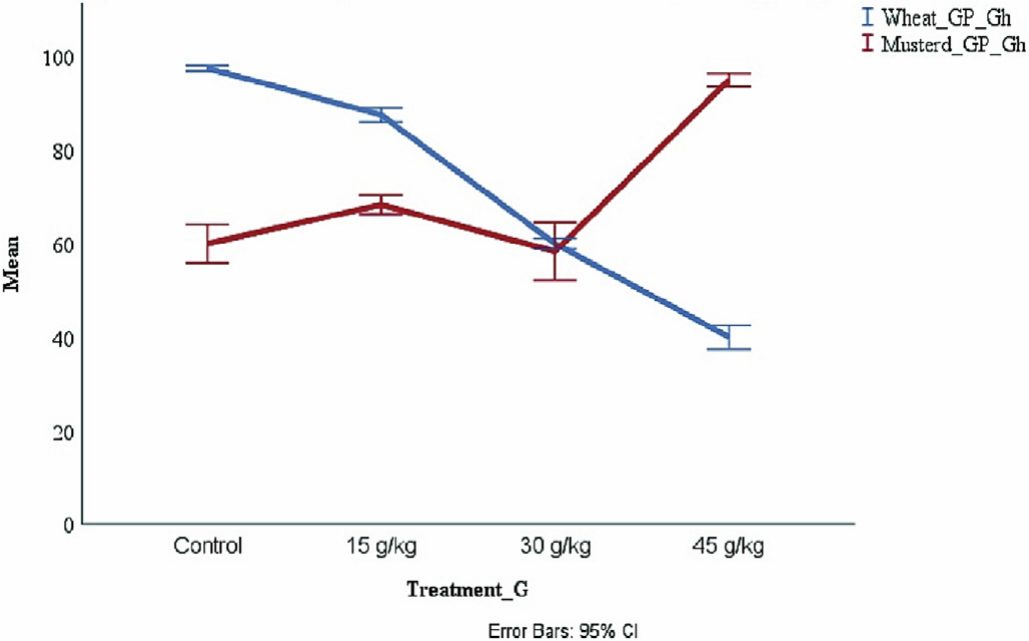
Mean of Wheat, and Mustard germination percentage (GP) in a greenhouse under different Treatments.

The seedling measurements of (GP, SL, and RL) were recorded weekly in three weeks. The results concluded that the growth of Wheat and Mustard plants, as indicated by germination percentage (Table 4, Table 5), showed opposite outcomes with Wheat (Stimulatory effect); the most significant germination occurred at high concentration (45 g kg^−1^) (Fig. 2).

**Table 4:** Means of germination percentage, plant characteristics, and their standard deviations of wheat as influenced by different concentrations of *Casuarina* dried leaf powder.

Treatment	GP	±SD	SL	±SD	RL	±SD
Control	97.50	0.50	5.28	0.33	5.36	0.52
15 g/kg	87.50	1.50	5.16	0.12	3.75	0.42
30 g/kg	60.00	1.15	4.42	0.19	3.50	0.44
45 g/kg	40.00	2.50	4.22	0.21	3.37	0.53

**Table 5:** Means of germination percentage, plant characteristics, and their standard deviations of Mustard as influenced by different concentrations of *Casuarina* dried leaf powder.

Treatment	GP	±SD	SL	±SD	RL	±SD
Control	60.00	4.08	9.60	4.22	4.08	0.74
15 g/kg	68.30	2.06	3.75	0.65	3.37	0.62
30 g/kg	58.33	6.13	3.79	0.57	3.33	0.98
45 g/kg	95.00	1.50	5.75	1.30	2.37	1.41

A significant variation of seed germination percentage, shoot, and root length of wheat and Mustard between treatments were found except the Mustard root length had no differences among means (*p* > *0,05*) at 95% confidence interval (Table 6).

**Table 6:** Analysis of variance of seed germination percentage, shoot, and root length of wheat and Mustard as influenced by different concentrations of *Casuarina* leaf extracts of Wheat plant in the greenhouse.

ANOVA		df	Mean Square	F	Sig.	ANOVA		df	Mean Square	F	Sig.
Wheat (GP)	Between Groups	3	2741.6	**1074.5**^a^	.000	Mustard (GP)	Between Groups	3	1151.22	**75.952**^a^	.000
	Within Groups	12	2.55				Within Groups	12	15.16		
	Total	15					Total	15			
Wheat (SL)	Between Groups	3	1.12	**24.39**^a^	.000	Mustard (SL)	Between Groups	3	30.22	**6.067**^a^	0.009
	Within Groups	12	0.046				Within Groups	12	4.98		
	Total	15					Total	15			
Wheat (RL)	Between Groups	3	3.41	**14.68**^a^	.000	Mustard (RL)	Between Groups	3	1.971	2.015	0.166
	Within Groups	12	0.23				Within Groups	12	0.979		
	Total	15					Total	15			

a Indicates significant differences among means at 0.05. Germination percentage (GP), shoot length (SL), root length (RL).

Two physical parameters (pH and EC) of soil were tested under the effect of *Casuarina* dry leaves that were mixed with soil in different concentrations (Table 7).

**Table 7:** Means of pH, the electrical conductivity of soil, and their standard deviations as influenced by different concentrations of *Casuarina* leaf extracts of wheat and Mustard experiment in a greenhouse.

Treatment	pH	±SD	EC	±SD
Control	8.50	0.20	659.5	108.20
15 g/kg	8.40	0.42	709.5	26.60
30 g/kg	7.71	0.20	1054	156.25
45 g/kg	7.81	0.30	1154	233.39

**Fig. 3:**
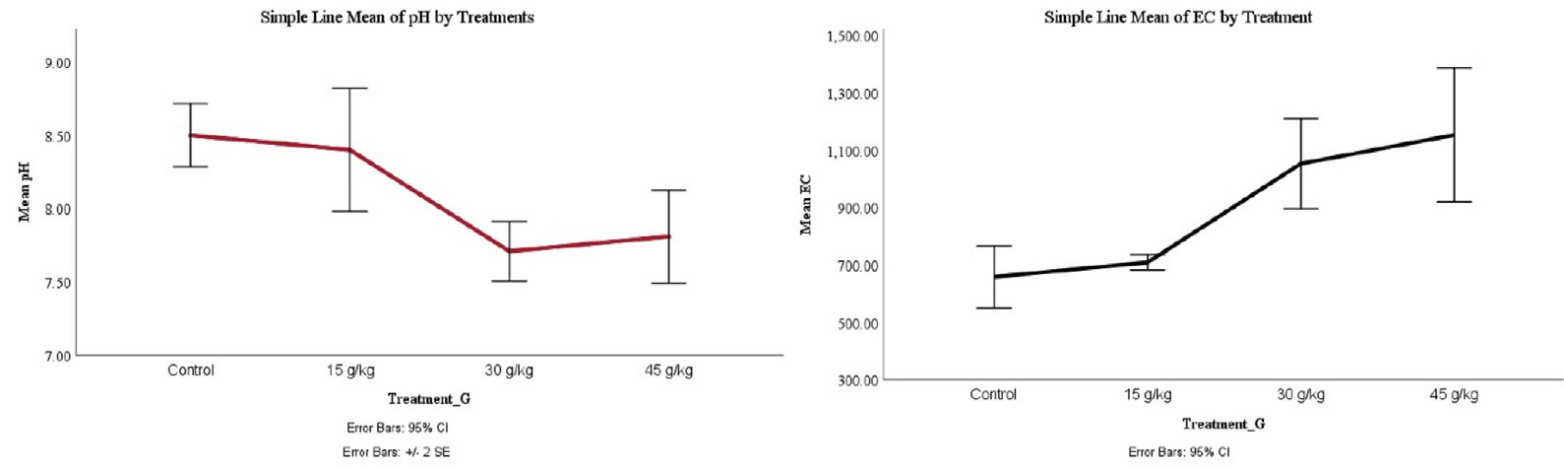
A-line chart of pH and EC under the effect of four different treatments.

The mean of measured pH values of soil was decreased gradually with increasing the treatment concentration, while the mean of EC was on the contrary (Fig. 3).

ANOVA test represents a noted variation (*p* > *0.05*) between treatments in both pH and EC parameters. (Table 8).

**Table 8:** Analysis of variance of pH and electrical conductivity of soil as influenced by different concentrations of *Casuarina* leaf extracts of Wheat and Mustard experiment in a greenhouse.

ANOVA		df	Mean Square	F	Sig.
pH	Between Groups	3	.648	**7.10**^a^	.005
	Within Groups	12	.091		
	Total	15			
EC	Between Groups	3	242973.67	**10.66**^a^	.001
	Within Groups	12	22784.62		
	Total	15			

a Indicates significant differences among means at 0.05. The potential of Hydrogen (pH) and Electrical conductivity (EC).

## 2 Experimental design, materials, and methods

The treatments were organized in a completely randomized design (CRD) with four replicates in each set. In the Petri dish experiment, each single petri dish was considered as a replicate. Moreover, in the greenhouse experiment, each pot is considered one replicate

### 2.1 Preparation of aqueous extract of *Casuarina equisetifolia*-L

**Fig. 4:**
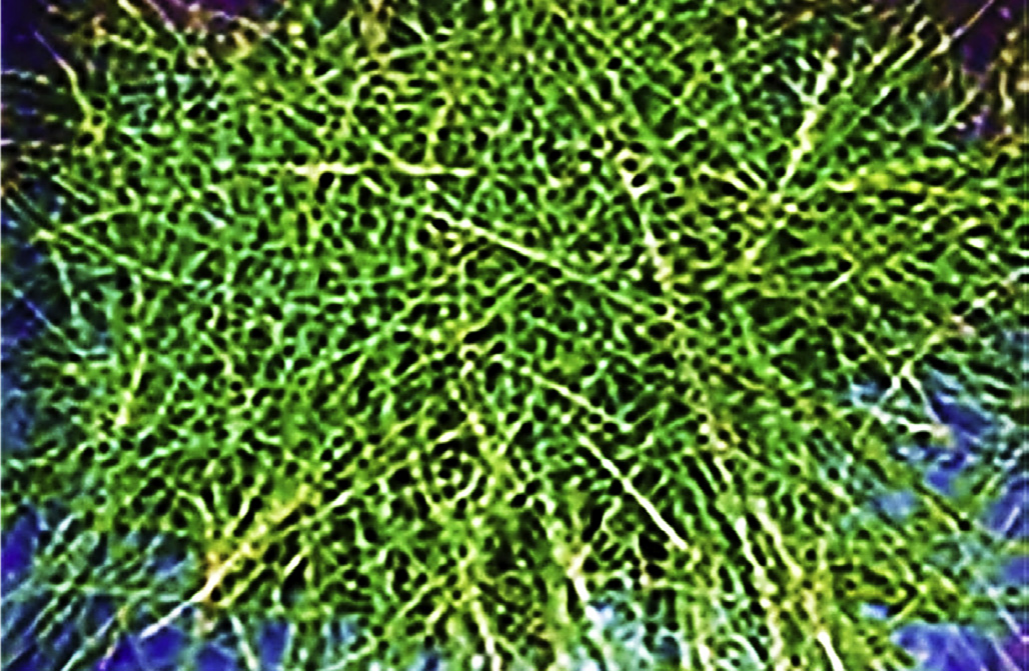
Collection of *Casuarina equisetifolia*-L. leaves.

Fresh leaves of *Casuarina equisetifolia* were collected from the area surrounding the College of Arts and Sciences, Qatar University. Leaves from mature trees were brought to the laboratory and were air-dried in the oven at 50 °C for 48 hours (Fig. 4). Then, the dried leaves were chopped into small pieces and ground into a fine powder. Distilled water was used to make plant extraction. Approximately 5 g of dried leaves powder was soaked into 50 ml distilled water followed by vigorous shaking for 48 hours at room temperature. The resulting solution was centrifuged, and the supernatant was filtered using a vacuum filter unit 1000 ml (Buchner Apparatus) to obtain a stock solution. The resulting brownish and dark extract was stored in the fridge in conical flasks until required.

**Fig. 5:**
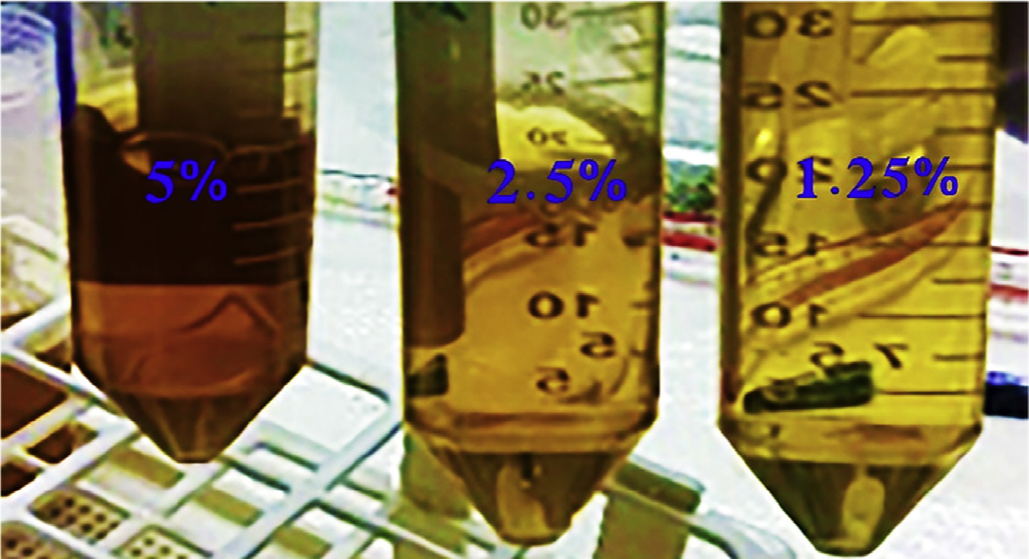
Three different extract concentrations 1.25%, 2.50%, and 5% obtained from the stock solution of *C. equisetifolia* Leaves.

Sequential dilutions were applied to the prepared stock solution to obtain three different concentrations (1.25%, 2.50%, and 5.00%) using Equation (1) (Table 9), with distilled water used as a control treatment (Fig. 5).

(1) $$DF\%=\frac{Initial\;volume}{Final\;volume}\times 100$$

where.

**Table 9:** Preparation of the different concentrations.

Volume taken (ml)	Volume up to (ml)	Final concentration (DF%)
12.5	1000	1.25
25	1000	2.5
50	1000	5

DF% is a final concentration percentage after dilution.

### 2.2 Germination test

**Fig. 6:**
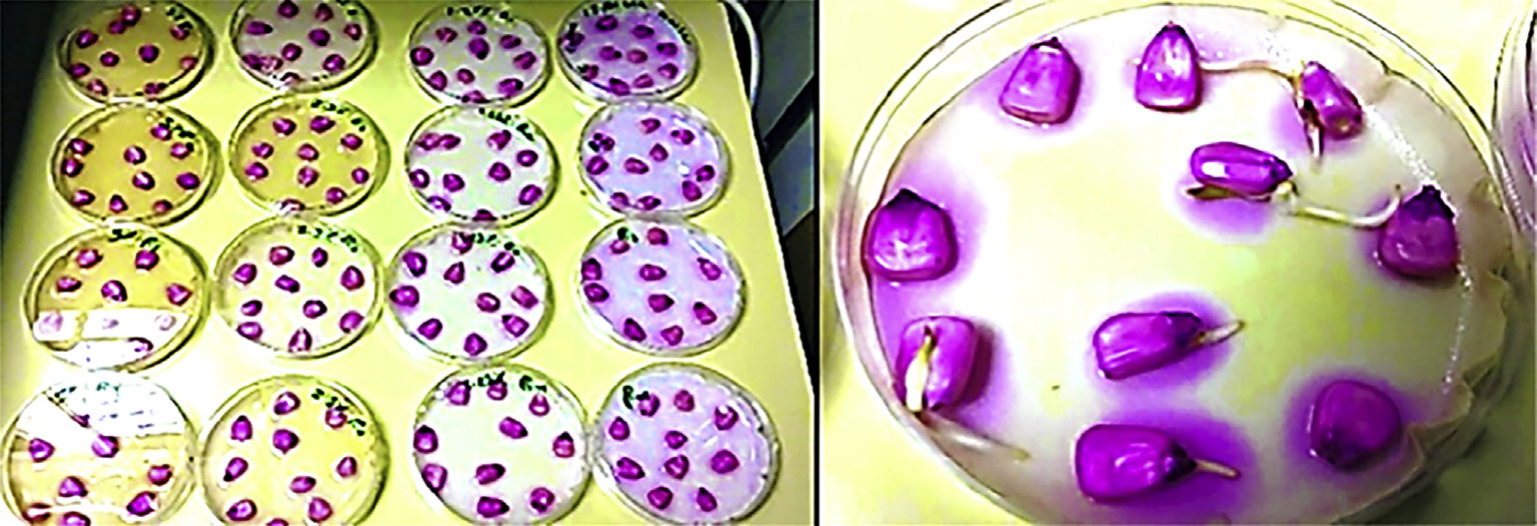
Germination test on Maize seeds by different extract concentrations.

Seeds of four crop plants (Maize, lentil, Mustard, and Wheat) were used in the seed germination test. Healthy uniform size seeds were selected and washed thoroughly four times by distilled water to remove any chemical residues and well dried using tissue paper. 10–20 seeds of each species were placed evenly (based on seed size) in sterilized 9 cm Petri dishes lined with a double layer of Whatman filter papers. The experimental set includes four different concentration of aqueous extract 0% 1.25%, 2.50% and 5.00%. The treated Petri dishes with distilled water were taken as a control, and it was considered as a zero concentration, while others were treated with approximately 8 ml of different aqueous extracts. Petri dishes were kept undisturbed under controlled lab conditions (room temperature (24 ± 2 °C), light) on the laboratory bench until germination occurs. (Fig. 6).

### 2.3 Seed germination and seedling growth

Seeds are considered germinated upon radical emergence, germination is determined by counting the number of germinated seeds each two days interval over a 6–8 days period to observe the germination rate. After eight days, the germination percentage (GP) was determined by counting the number of seeds germinated during the period of the experiment over the total number of seeds based on Equation (2), [5].

(2) $$GP=\frac{Number\;of\;germinated\;seeds}{Total\;number\;of\;seeds}\times 100$$

Where GP. Germination percentage.

Shoot length and root length were measured using a centimeter ruler by taking five seedlings at random from each petri dish.

### 2.4 Greenhouse experiment

**Fig. 7:**
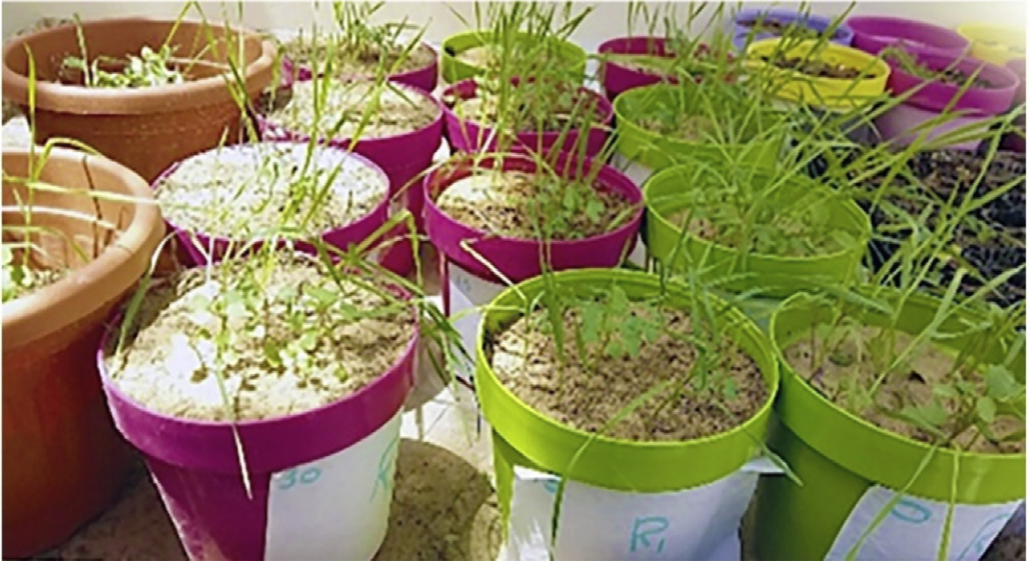
Effect of incorporation of *C. equisetifolia* leaf powder on Wheat and Mustard (weed) germination and growth.

The experiment was performed in the greenhouse to investigate the effect of *C. equisetifolia* leaves on plant growth. Two different crop plants were selected to grow together in this experiment under *C. equisetifolia* leaf residuals with different dosages. Mustard is considered as a weed plant and competition factor against wheat growth. Dried leaf powder of *C. equisetifolia* was mixed with peat moss and clay (1:4) at a rate of 15, 30, and 45 g kg^−1^ of soil, in addition to the control treatment with no leaf powder. Plastic pots of 15 cm in diameter were filled with 2 kg of soil. Ten viable seeds of wheat with fifteen seeds of Mustard were sown together in each pot. Water supplied as required to avoid water stress. Pots were arranged in a completely randomized block design with four replications (Fig. 7).

### 2.5 Data collection of the greenhouse experiment

Number of germinated seeds was observed weekly in three weeks, then germination percentage was calculated by counting the number of seeds germinated over the total number of seeds based on Equation (2), [5]. After plant maturity, data were recorded for growth parameters including the number of shoots, root lengths (cm), number of spikes, plant height, fresh and dry weights (g/plant), stem diameter (mm), as well as a number of leaves. In addition, soil physical analysis was conducted Electric conductivity EC and pH. In the end, statistical analysis was conducted to all the collected results using analysis of variance and means comparisons.
